# RNA-Seq Revealed Expression of Many Novel Genes Associated With *Leishmania donovani* Persistence and Clearance in the Host Macrophage

**DOI:** 10.3389/fcimb.2019.00017

**Published:** 2019-02-05

**Authors:** Mohammad Shadab, Sonali Das, Anindyajit Banerjee, Roma Sinha, Mohammad Asad, Mohd Kamran, Mithun Maji, Baijayanti Jha, Makaraju Deepthi, Manoharan Kumar, Abhishek Tripathi, Bipin Kumar, Saikat Chakrabarti, Nahid Ali

**Affiliations:** ^1^Infectious Diseases and Immunology Division, Indian Institute of Chemical Biology, Kolkata, India; ^2^Structural Biology and Bio-Informatics Division, Indian Institute of Chemical Biology, Kolkata, India; ^3^Nucleome Informatics Pvt. Ltd., Hyderabad, India

**Keywords:** transcriptome, RNA seq, macrophage, signaling/signaling pathways, *Leishmania donovani*

## Abstract

Host- as well as parasite-specific factors are equally crucial in allowing either the *Leishmania* parasites to dominate, or host macrophages to resist infection. To identify such factors, we infected murine peritoneal macrophages with either the virulent (vAG83) or the non-virulent (nvAG83) parasites of *L. donovani*. Then, through dual RNA-seq, we simultaneously elucidated the transcriptomic changes occurring both in the host and the parasites. Through Kyoto Encyclopedia of Genes and Genomes (KEGG) pathway analysis of the differentially expressed (DE) genes, we showed that the vAG83-infected macrophages exhibit biased anti-inflammatory responses compared to the macrophages infected with the nvAG83. Moreover, the vAG83-infected macrophages displayed suppression of many important cellular processes, including protein synthesis. Further, through protein-protein interaction study, we showed significant downregulation in the expression of many hubs and hub-bottleneck genes in macrophages infected with vAG83 as compared to nvAG83. Cell signaling study showed that these two parasites activated the MAPK and PI3K-AKT signaling pathways differentially in the host cells. Through gene ontology analyses of the parasite-specific genes, we discovered that the genes for virulent factors and parasite survival were significantly upregulated in the intracellular amastigotes of vAG83. In contrast, genes involved in the immune stimulations, and those involved in negative regulation of the cell cycle and transcriptional regulation, were upregulated in the nvAG83. Collectively, these results depicted a differential regulation in the host and the parasite-specific molecules during *in vitro* persistence and clearance of the parasites.

## Introduction

Macrophages are known to have microbicidal functions and are considered as the sentinels of the immune system (Franken et al., 2016). However, their interaction with pathogens (of high and low virulence) varies significantly. Virulent pathogens avert the antimicrobial functions of the macrophages to survive and persist, while the less virulent ones are unable to exhibit the same and thus get eliminated (Chakrabarty et al., 1996; Olivier et al., 2005). During this host-pathogen interaction, global changes in the gene expression pattern occur, both in the host (macrophages) as well as the infecting pathogens. If we could identify those genes, they could serve as tools to develop potent antimicrobial interventions. In this study, we attempted to identify the host as well as the parasite-specific genes, which were modulated when the host macrophages interacted with the virulent and the non-virulent *L. donovani* parasites (vAG83 and nvAG83, respectively) (Sinha et al., 2018). To obtain nvAG83 parasites, we first cultured the vAG83 for several passages in medium, and then performed genomic and transcriptomic studies on both the early passaged vAG83 and the late passaged nvAG83 parasites (Sinha et al., 2018). With these two parasites, we infected the non-elicited murine peritoneal macrophages (Ghosn et al., 2010), and measured the transcriptome of both the host as well and the infecting parasites with high-throughput deep sequencing (RNA-Seq) technology. RNA-Seq ensures a highly sensitive technique with high accuracy and provides a far more precise measurement of the level of transcripts than most other methods (Wang et al., 2009).

Numerous other studies have elucidated the host cell gene expression in response to *Leishmania* infection using microarray analysis (Probst et al., 2012; Ovalle-Bracho et al., 2015). One such study compared the gene expression in macrophages infected by two different *Leishmania* parasites (*L. donovani* and *L. major*) (Gregory et al., 2008). Another study compared the effect of a single strain of the *Leishmania* parasite (*L. amazonensis*) on the gene expression of two different macrophages that were isolated from the peritoneal cavity of C57BL/6 and CBA mice (Probst et al., 2012). Reports on the gene expression study, in the context of both the host as well as the infecting *Leishmania* parasites, are limited. There is a study using serial analysis of gene expression (SAGE), which has simultaneously analyzed gene expression patterns in human macrophages and the infecting *L. major* parasites (Guerfali et al., 2008). However, due to the limitations associated with this tag-based sequencing technique, it is difficult to achieve a comprehensive gene expression profiling (transcriptome) of both the interacting subjects in question (the host and the parasites). However, with the newly-developed RNA-Seq technology, these limitations have been overcome quite convincingly (Wang et al., 2009). Recently, with RNA-Seq, simultaneous transcriptional profiling of *L. major* and its host macrophages was done to understand how virulent parasites could evade host responses in order to survive in the mammalian environment (Dillon et al., 2015). These studies, however, did not address changes in gene expression, when the host cells kill non-virulent parasites.

Simultaneous gene expression studies in macrophages infected with *L. donovani* parasites have not been done so far. Moreover, though the gene expression analysis in macrophages infected with vAG83 (a virulent strain) has been reported through microarray analysis (Buates and Matlashewski, 2001), such studies in macrophages infected with nvAG83 (a non-virulent strain) have also not been evaluated so far. Therefore, the focus of our study was to unravel host as well as parasite-specific genes that were modulated when vAG83 persists and nvAG83 gets eliminated in the host macrophages.

Through KEGG pathway and gene ontology analyses, we discovered a significant difference in the host responses evoked by the vAG83 and the nvAG83 parasites. It was found that vAG83 induces an immunosuppressive condition, whereas nvAG83 induced an immune-stimulatory environment within the host cells. In these two parasite-infected macrophages, we also found that the protein-protein interactome was altered differentially. While vAG83 downregulated, nvAG83 upregulated the expression of many hub and hub-bottleneck genes in the host macrophages. Further, the mitogen-activated protein kinase (MAPK) and Phosphatidylinositol 3-kinase (PI3K) signaling pathways were also modulated differentially. It was noted that vAG83 induced higher activation of ERK1/2 and AKT (ser437) in the host macrophages as compared to nvAG83. Conversely, nvAG83 induced higher activation of P38 in the host macrophages as compared to vAG83. A differential gene expression pattern was also observed in the two infecting parasites. Gene expression analyses showed that the genes related to virulence and survival were significantly overexpressed in vAG83 as compared to nvAG83. In contrast, the immunostimulatory genes and negative regulators of cell survival machinery were significantly overexpressed in nvAG83 as compared to vAG83. Thus, this work provided valuable insights into how the host responses could become modulated upon infection with a virulent and a non-virulent AG83 parasite. Moreover, it also provides good insights into the responses generated in these two infecting parasites. Overall, this study deciphered various important factors of the host as well as the parasites, which are significantly associated with either the parasite's survival or clearance in the host macrophages.

## Materials and Methods

### Animals and Parasites

BALB/c mice, bred in the animal house facility of the Indian Institute of Chemical Biology (Calcutta, India), were used for the experiments*. L. donovani* strain AG83 (MHOM/IN/1983/AG83), originally isolated from an Indian kala azar patient, was maintained by serial passage in 4- to 6-week-old Syrian golden hamsters (*Mesocricetus auratus*) reared in a pathogen-free animal care facility of the Indian Institute of Chemical Biology. Animal monitoring was done as per the Animal Ethics Committee approved protocol. Animals were not sedated for any procedures. The animals were euthanized for isolating *L. donovani* amastigotes periodically as per the approved animal ethics of our institute (147/1999/CPSCEA). These amastigotes were then transformed into promastigotes in Schneiders's medium supplemented with 20% heat-inactivated FCS and penicillin G (100 U/ml) and streptomycin sulfate (100 μg/ml), and then the culture was maintained in M199 supplemented with 10% FCS, 2 mM glutamine, penicillin G (100 U/ml) and streptomycin sulfate (100 μg/ml) at 22°C by weekly passaging up to the 25th passage. The early passaged parasites (2nd) were considered as virulent and the late passaged (25th) were considered as non-virulent based on infection study.

### Preparation of Peritoneal Macrophages and Infection

BALB/c mice, bred in the animal house facility of the Indian Institute of Chemical Biology (Calcutta, India), 8–10 weeks old were used for the experiments. By cervical dislocation mice were sacrificed. Their peritoneal macrophages were then collected by infusing the peritoneal cavity with ice-cold sterile RPMI supplemented with 3% FCS. Cells (1 × 10^6^) were then dispensed into 6-well plates and allowed to adhere overnight at 37°C in 5% CO_2_. Non-adherent cells were washed off, and wells were replenished with RPMI 1640 containing 10% FCS. Cells were infected at a parasite-to-macrophage ratio of 10:1 for 3 h at 37°C in a humidified atmosphere of 5% CO_2_, after which non-ingested promastigotes were washed off with warm RPMI and incubated for the indicated time point. The cells were then lysed in trizol for RNA isolation. RNA samples from two independent experiments were pooled together for poly (A)-enriched cDNA preparation.

### *In vitro* Infection

For *in vitro* infection, 2 × 10^5^ peritoneal macrophages were cultured on cover slips and incubated overnight. The next day coverslips were washed with warm RPMI medium to remove the unattached cells and then infected at a 1:10 ratio with the parasites. After 3 h of incubation, the uningested parasites were removed by washing three times with the RPMI medium. The infected cells were then incubated for various time points post-infection. The coverslips were then washed twice with PBS and air-dried. After methanol fixation, the cells were Giemsa-stained and the number of amastigotes in 100 macrophage cells was counted under a light microscope using oil emersion lenses.

### Infection to Animals and Determination of Splenic and Hepatic Parasite Burden

BALB/c mice (4–5 weeks) were infected by injecting 2 × 10^7^ stationary phase promastigotes as described above. Mice were sacrificed at 12 weeks post-infection for determination of parasite burden.

#### Leishman-Donovan Units (LDU)

Spleen and liver were removed at specified times, and multiple impression smears were prepared and stained with Giemsa (Banerjee et al., 2008). Organ parasite burdens, expressed as LDU, were calculated as the number of parasites per 1,000 nucleated cells × organ weight (in mg).

#### Limiting Dilution Assay (LDA)

To further evaluate whether the spleen and liver contained live parasites, the parasite burden was quantified in these tissues by serial dilution assay (Banerjee et al., 2008). Briefly, a weighed piece of spleen or liver from experimental mice was first homogenized in Schneider's medium supplemented with 10% FCS, and then diluted with the same medium to a final concentration of 1 mg/ml. Five-fold serial dilutions of the homogenized tissue suspensions were then plated in 96-well plates and incubated at 22°C for 21 days. Wells were examined for viable and motile promastigotes at 7-day intervals, and the reciprocal of the highest dilution that was positive for parasites was considered to be the parasite concentration per mg of tissue. The total organ parasite burden was calculated using the weight of the respective organs.

### RNA Isolation and cDNA Library Preparation

Total RNA was isolated using the Trizol® reagent (Invitrogen, CA), treated with DNase and purified using the Qiagen RNeasy mini kit. Quality checks of the RNA samples were performed with Qubit (picogreen) to assess sample concentration. Subsequent steps were followed according to the protocols prescribed by Illumina (Cat# RS-930-1001). Poly (A)-enriched cDNA library preparation involved purifying the poly-A-containing mRNA molecules from total RNA using oligo-dT-attached magnetic beads. Following purification, the mRNA is fragmented into small pieces using divalent cations under elevated temperature. The cleaved RNA fragments are copied into first-strand cDNA using reverse transcriptase and random primers. Second-strand cDNA synthesis followed, using DNA PolymeraseI and RNase H. The cDNA fragments then went through an end repair process, the addition of a single “A” base, and then ligation of the adapters. The products were then purified and enriched with PCR to create the final cDNA library.

### Sequencing

Sequencing of both QC passed libraries was performed on the Illumina HiSeq2500 system. The HiSeq 2500 system is a powerful and efficient ultra-high-throughput sequencing system that supports the broadest range of applications and study sizes (Reuter et al., 2015). Unrivaled data quality using Illumina's proven SBS Chemistry (Ambardar et al., 2016) have made the HiSeq2500 the instrument of choice for Molecular Biologists (Table 1).

**Table 1:** Sequencing summary.

**INSTRUMENT**	
Manufacturer	Illumina
Version	HiSeq 2500
Slot used	A
Basecalling pipeline	-HiSeq Control Software 2.2.38 -RTA 1.18.61.0 -CASAVA-1.8.2
**RUN**	
Mode	HiSeq high output (HO) version 4
Number of cycles	2 × 150 + 7
Number of lanes	1
Flow cell ID	HL7FHCCXX
Flow cell version	HiSeq Flow Cell v4
Kit version	HiSeq SBS Kit v4
Indexing	Single-Indexing
Data output C	5.18 Gb
Data output (nvAG83 infected macrophage)	4.36 Gb
Data output (vAG83 infected macrophage)	4.36 Gb
**SEQUENCING SPECIFICATION**	
Service	Full lane
Error rate	1.5%
Q30	80%

### RNA-Seq Data Generation, Pre-processing and Quality Trimming

The next-generation sequencing run for whole transcriptome sequencing was performed using the paired-end (PE) 2 × 150 bp library on the Illumina HiSeq 2500. Raw data were generated for each of the libraries from the three samples (Supplementary Table 1). Trimmomatic version-0.33 was used for pre-processing of raw reads generated for the samples. Parameters considered for filtration were as follows: ADAPTER TRIMMING: 2:30:10. SLIDINGWINDOW: 4:20. MINLENGTH: 50. For paired-end data, two input files were specified to Trimmomatic, and Trimmomatic produced 4 output files, 2 for the “paired/PE” output where both reads survived the processing, and 2 for corresponding “unpaired/single-end/SE” output where a read survived, but the partner read did not. Thus, the total clean reads obtained for each sample was calculated as PEx2+SE. Sequence quality metrics were assessed using FastQC (version: 0.11.3) (http://www.bioinformatics.babraham.ac.uk/projects/fastqc/).

### Mapping cDNA Fragments to the Reference Genome, Read Count and Data Normalization

Clean reads from each sample were aligned independently to the *Mus Musculus* (UCSC mm10) reference genome and *L. donovani* (LdBPK282A1) reference genome using TopHat 2 (parameter: -g 1 and default) (Trapnell et al., 2009). TopHat is a fast splice junction mapper for RNA-Seq reads. It aligns RNA-Seq reads to mammalian-sized genomes using the ultra-high-throughput short read aligner Bowtie, and then analyzes the mapping results to identify splice junctions between exons. The HTSeq tool was used to count the number of reads aligned to protein coding genes. HTSeq is a Python package that provides infrastructure to process data from high-throughput sequencing assays.

### Differential Expression Analysis

The DESeq tool was used for differential gene expression analysis between samples in protein coding genes. The DESeq is an R package to estimate variance-mean dependence in count data from high-throughput sequencing assays and testing for differential expression based on a model using the negative binomial distribution. Differentially expressed (DE) genes were defined as genes with a Benjamini-Hochberg multiple testing *p* value of <0.05.

### KEGG Pathway Analysis

ConsensusPathDB-mouse was done to identify signaling and metabolic pathways that were over-represented in the mouse DE gene lists. KOBAS was used to find the enrichment analysis in KEGG and Gene Ontology. Both *p* < 0.05 and paj/FDR value < 0.05 genes were used for KEGG enrichment analysis. KOBAS can identify statistically significantly enriched pathways, human diseases, and functional terms for an input set of genes using biological knowledge from well-known pathway databases, disease databases, and gene ontology. The Hypergeometric test and Fisher's exact test were used for statistical testing in enrichment analysis and Benjamini-Hochberg was used for the FDR correction method. For each KEGG pathway, a *P-*value was calculated using a hypergeometric test, and a cutoff of 0.01 was applied to identify enriched KEGG pathways. Genes that were DE more than 2-fold in *L. donovani*-infected cells relative to uninfected controls were used as input, with up- and down-regulated genes considered separately. For generating heat maps of these genes, in-house script software was used.

### Gene Ontology (GO) Analysis

GO categories enriched in the *L. donovani* DE gene lists were identified using the GOseq package in R. It detects gene ontology and/or other user-defined categories that are over/under-represented in RNA-Seq data. Gene ontology analysis was used for RNA-Seq and other length-biased data. For each comparison, upregulated and downregulated gene sets (no fold change cut-off) were input separately into GOseq. A *p-*value cut-off of 0.05 was used. The hypergeometric method was used for enrichment analysis.

### Isolation of Total RNA, cDNA Synthesis and Real-Time PCR

Total RNA from macrophage cells (uninfected and infected with both parasites) was isolated using Trizol reagent (Invitrogen), according to the manufacturer's instructions. A total of 2 μg of RNA from each sample was reverse-transcribed to cDNA using the iScript cDNA synthesis kit (Bio-rad), according to the manufacturer's protocol. Using LightCycler 96 (Roche), real-time PCR was performed according to the SYBER GREEN method (KAPA BIOSYSTEMS). The PCR thermocycling parameters were kept as 95°C for 10 min, 45 cycles of 95°C for 15 s, 55°C for 30 s and 72°C for 25 s. GAPDH was used as an internal control. Samples were run in duplicates. The fold induction was determined by the 2^−ΔΔ*CT*^ method (Schmittgen and Livak, 2008). Fold induction of duplicate samples were averaged.

### Construction of Protein-Protein Interaction of the Deregulated Genes

Protein-protein interactions (PPI) of the virulent and non-virulent specific macrophage genes were searched in the STRING database (von Mering et al., 2003). Interactions with the highest confidence values [experimental evidences score ≥ 0.9] were collected to construct the network, which was further used for network topology analysis for identifying important interactive nodes of the network. The Hub Objects Analyzer (Hubba) (Lin et al., 2008) is a web-based plug-in incorporated in Cytoscape (Shannon et al., 2003) was used to determine the important network nodes—i.e., the hub (He and Zhang, 2006) and bottleneck (Yu et al., 2007; McDermott et al., 2009).

### Preparation of Bone Marrow-Derived Macrophages (BMM)

To generate bone marrow-derived macrophages (BMM), uninfected BALB/C mouse femurs were aseptically harvested and sterilized in 70% (vol/vol) ethanol for 1 min and then washed thrice in Dulbecco's phosphate-buffered saline (PBS). The contents of the femurs were flushed out with complete DMEM using a 25-guage needle. Bone marrow cells were then cultured and differentiated in 35 mm cell culture dishes containing DMEM supplemented with 100 U/ml penicillin-streptomycin, 10% fetal bovine serum, and 10 ng/ml MCSF for 7 days at 37°C in a humidified atmosphere of 5% CO_2_.

### Western Blotting

Macrophages (1–2 × 10^6^) cultured in 35 mm cell culture dishes were infected with promastigotes of *L. donovani* at a 1:10 ratio for different time points. Cells were then washed with PBS and lysed in cell lysis buffer containing protease and phosphatase inhibitor cocktail, and the protein concentrations in the cleared supernatants were estimated using Lowry's method. The cell lysates were resolved by 10% SDS-PAGE and then transferred to Nitrocellulose membranes (BioRad). The membranes were blocked with 5% BSA in Tris-buffered saline (TBS) for 1 h at room temperature and probed with primary Ab for 2 h at a dilution recommended by the manufactures. Membranes were then washed three times with wash buffer (TBS containing 0.5% tween) and then incubated with HRP-conjugated secondary Ab and detected by an ECL detection system according to the manufacturer's instructions.

### Statistical Calculations

All data comparisons were tested for significance with the two-tailed Student's *t*-test using GraphPad software; *P-*values < 0.05 were considered significant.

## Results and Discussion

### Infection Dynamics and the Global Transcriptional Changes in Macrophages Infected With vAG83 and nvAG83 Parasites

**Figure 1:**
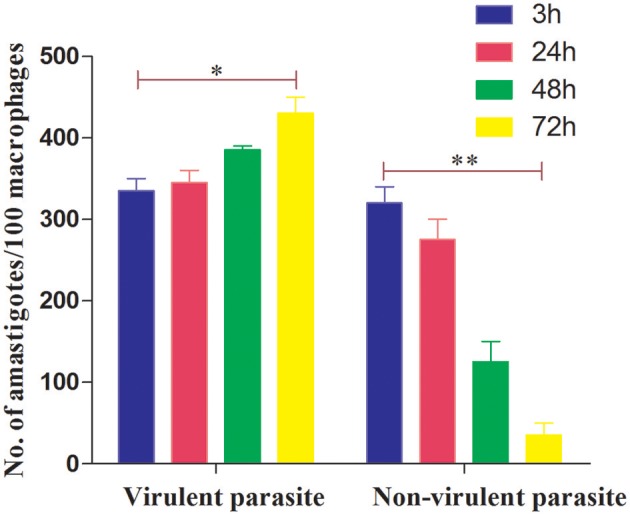
Dynamics of murine macrophage infection with virulent and non-virulent *L. donovani* promastigotes. Murine macrophages were infected with either virulent (2nd passage) or non-virulent (25th passage) *L. donovani* promastigotes for 3 h, washed, and further incubated until 24, 48, and 72 h p.i. The number of internalized parasites in 100 macrophages was determined microscopically by Giemsa staining. The results are expressed as mean S.E (*n* = 3). ^*^P < 0.01, ^**^P < 0.001.

We first determined a time-dependent infection pattern in the murine peritoneal macrophages, co-cultured with the promastigotes of vAG83 (2nd passaged) and nvAG83 (25th passaged). Parasite load was estimated after 3, 24, 48, and 72 h post-infection (p.i.). It was observed that the initial parasite count at 3 h p.i. was comparable for both the parasites, although it was slightly lower for nvAG83. However, vAG83 exhibited a progressive increase in the parasite burden with time. nvAG83, in contrast, showed a time-dependent decrease in parasite load (Figure 1). To validate these *in vitro* results in an *in vivo* set-up, BALB/c mice were infected with promastigotes derived from different passages, and the parasite burden was determined in both the liver and the spleen, using methods like Leishman Donovan Units (LDU) and Limiting Dilution Assay (LDA). The *in vivo* infectivity of the parasites also declined when they were cultured in the medium repeatedly. The results of the LDU and the LDA revealed that, with an increasing number of parasite passage, there was a progressive suppression in liver as well as splenic parasite burden (Table 2). Thus, these results confirmed that early passaged parasites were potent enough to establish successful infection, but the late passaged ones were weak and unable to do so, both *in vitro* (in macrophages) as well as *in vivo* (in mice model). Previously, we have shown differences in the *in vivo* parasite burden in the spleen and liver of hamsters infected with the early and the late passage promastigotes (Sinha et al., 2018). We found that these differences were attributed to a differential expression of several virulence factors and cytoskeletal proteins in the parasites. At the genomic level, we observed subtle changes, mostly in defense-related, nutrient acquisition and signal transduction-related genes between the two passages. We also observed SNPs in ABC transporter and calpain-like cysteine protease genes, which are emerging as patho-adaptive factors in clinical isolates of *Leishmania* (Sinha et al., 2018).

**Table 2:** *In vivo* parasite burden in mice.

**Passage number**	**LDU ± S.E. (*****n*** **= 3–5/group)**		**log**_****10****_**parasite burden ± S.E. (*****n*** **= 3–5/group)**	
	**Liver**	**Spleen**	**Liver**	**Spleen**
2nd	1056 ± 117.9	602.1 ± 109.3	16.34 ± 2.4	12 ± 1.18
5th	945.1 ± 94.87	514.5 ± 74.51	14.23 ± 3.15	11.52 ± 2.08
15th	302.9 ± 54.30	78.78 ± 15.10	7.94 ± 1.54	5.17 ± 1.88
25th	54.83 ± 11.04	7.96 ± 3.069	3.28 ± 2.11	2.29 ± 1.76

*BALB/c mice were infected with 2 × 10^7^ L. donovani promastigotes of different passages as indicated in the table. At 12 weeks, p.i. mice were sacrificed and parasite burden of the liver and spleen were determined by LDU and LDA. Data represent mean ± S.E.M of 3–5 animals per group*.

Subsequently, to determine the global gene expression pattern in both the host and the parasites, we infected murine peritoneal macrophages with vAG83 and nvAG83 for 12 h and generated the transcriptomic data using RNA-Seq. This time point of infection was chosen based on our study on the *in vitro* macrophage infectivity, where we found that after this time point, the infection is directed toward progression for vAG83 and reduction for nvAG83. Progressive clearance of nvAG83 with time hinders the effort of getting an ample amount of *Leishmania*-specific RNA that could be analyzed for their change. A total of 12 h of infection allowed us to compare the transcriptomic changes between the vAG83 and the nvAG83 infective stages of the parasites. The bioinformatics analysis workflow is represented in Supplementary Figure 1. Raw data (reads) were generated for the three samples (uninfected macrophages, and macrophages infected with vAG83 and nvAG83) (Supplementary Table 1), which were further processed to give clean reads. Reads (paired end/PE plus unpaired/single-end/SE) with a sequence of 150 nucleotides were generated (see Material and Methods), which yielded a total of 89.19 million high-quality reads from the three samples (Supplementary Table 2). The infected samples consisted of a pool of mixed RNAs from the mouse macrophages and the *Leishmania* parasites. However, the reads generated for the whole sample can be mapped to the genome of the mouse and the parasite RNAs, respectively. But the possibilities of error in mapping the reads across the two species cannot be denied as well. This is mainly because mapping of reads to both the species genome from the same sample depends on several factors, including the use of random hexamer for reverse transcription of poly (A) RNA, which may not retain information contained on the DNA strand that is actually expressed (Mortazavi et al., 2008). Secondly, the size of the final fragment to be sequenced is crucial for proper sequencing and subsequent analysis. Longer reads improve mappable data and transcript identification, whereas short SE reads are normally sufficient for studies of gene expression levels in well-annotated organisms (Garber et al., 2011). Depth of sequencing is also crucial in detection and quantification of the transcript in a precise manner (Mortazavi et al., 2008). For example, 70 and 90% of regular RNA-Seq reads are expected to map to the mouse genome depending on the read mapper used (Dobin et al., 2013). Thus, we can say that the fraction of reads mapping to the mouse vs. parasite reference genomes depicted the proportion of RNA molecules from each source but without denying all these factors. The percentage of PE and SE reads mapping to the mouse genome was found to be 82.6 and 84%, respectively, for the uninfected macrophages (Supplementary Table 3). Likewise, the percentage of PE and SE reads mapping to the mouse genome was found to be 67 and 66%, respectively, in the macrophages infected with vAG83, and 65.2 and 66.4%, respectively, in the macrophages infected with nvAG83. Similarly, the proportion of parasite-specific PE and SE reads in vAG83-infected macrophages corresponded to 14.5 and 16.3%, respectively, and in nvAG83-infected macrophages, parasite-specific PE and SE reads corresponded to 16.6 and 18.3%, respectively (Supplementary Table 4). Similar observations for mouse and parasite reads were reported earlier (Dillon et al., 2015). The reason for not getting 100% reads mapping to mouse and parasite genomes in these studies is not well understood. It may be that due to the dynamicity of the transcriptional activity, probing the whole transcriptome at a particular time is not feasible to obtain reads that can map to 100% to the reference genome.

**Figure 2:**
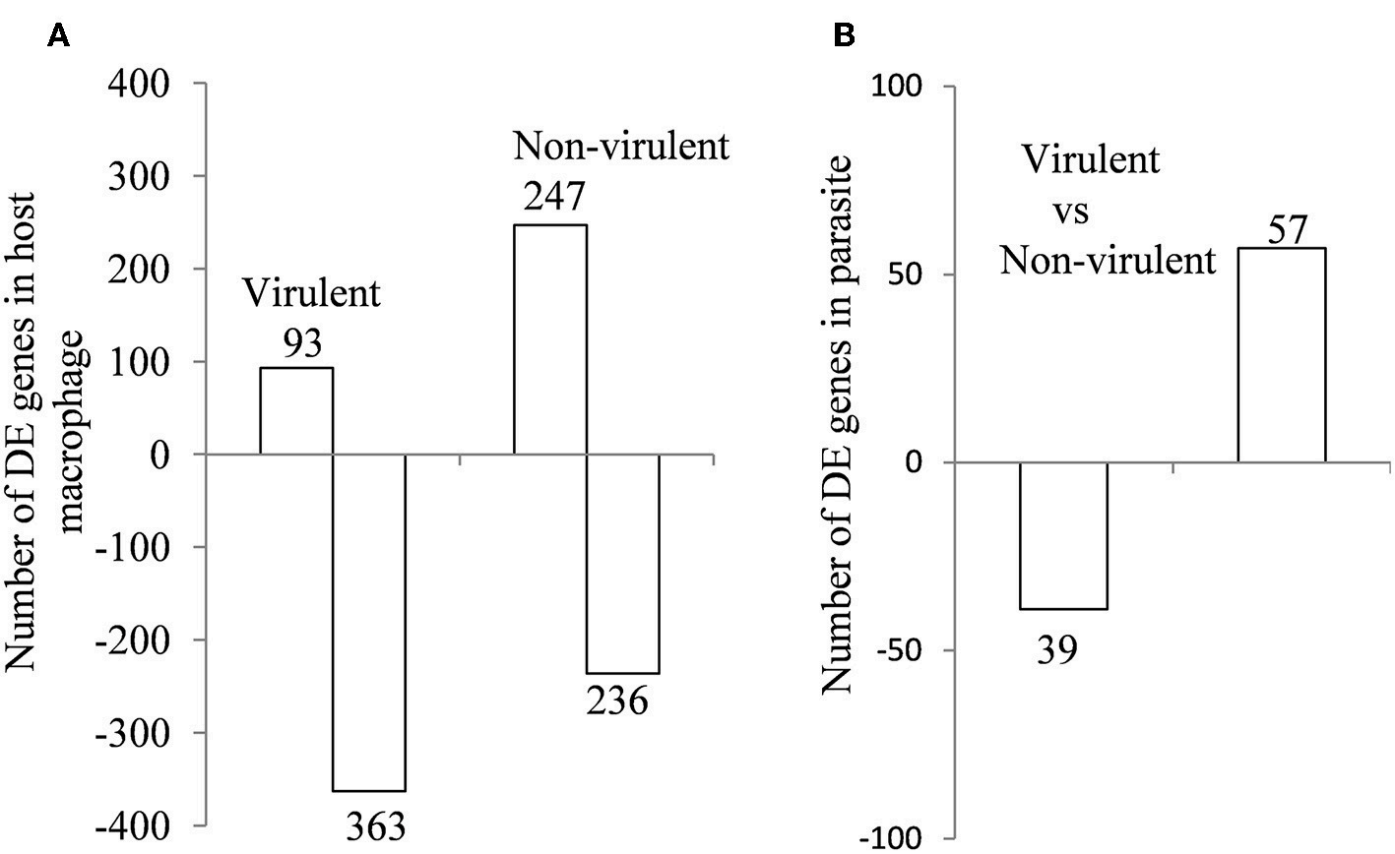
Differentially expressed genes in **(A)** murine macrophage infected with virulent and non-virulent *L. donovani* for 12 h and in **(B)** *L. donovani* parasites. The numbers of DE genes in *L. donovani*-infected macrophages relative to uninfected controls and *L. donovani* depicted as horizontal bar plots. Box length depicts the number of DE genes either downregulated or upregulated at a *P*-value of < 0.05 with the total number of down- and up-regulated genes shown.

**Figure 3:**
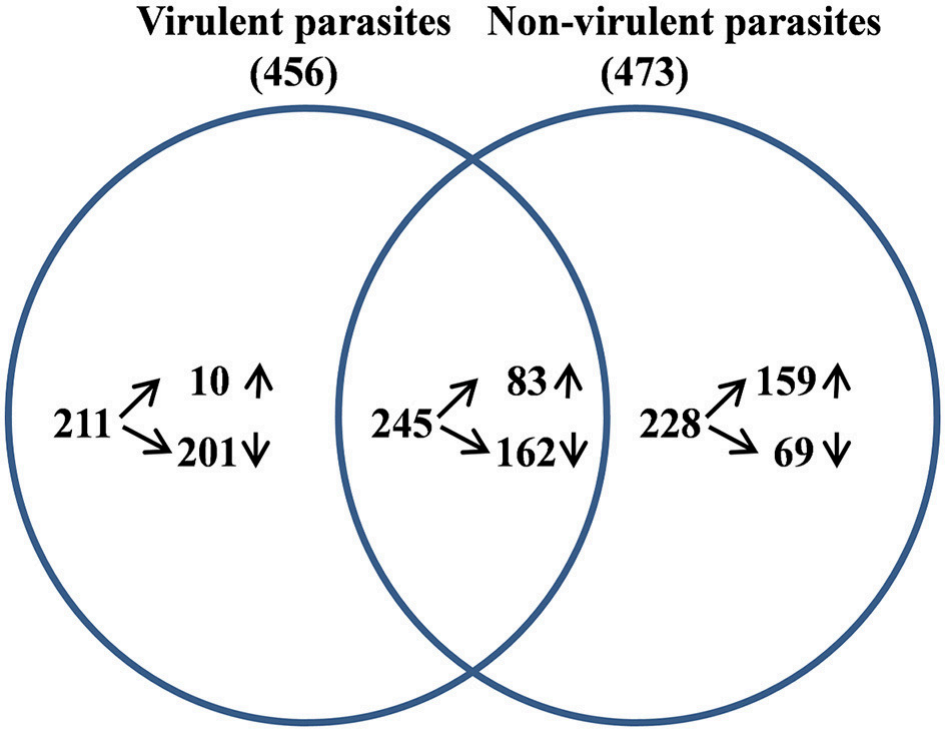
Analysis of differentially expressed (DE) genes in host macrophage at 12 h p.i. The DE gene lists for murine macrophage uninfected vs. infected with virulent and non-virulent parasites were compared and the overlap genes shown as a Venn diagram.

From the sequencing data set, we next identified differentially expressed (DE) genes in the infected macrophages as compared to the uninfected control. Thus, we prepared two gene lists, one for the macrophages infected with vAG83 (Supplementary Data Sheet 1A vAG83) and the other for the macrophages infected with nvAG83 (Supplementary Data Sheet 1B nvAG83). Moreover, we considered only those genes in the gene list, which were DE>2-fold, with upregulated and downregulated genes, considered separately. We found that in the host macrophages, vAG83 and nvAG83 induced 456 and 473 DE genes, respectively, that were differentially expressed at a *p*-value cutoff of < 0.05 compared to the uninfected control. Intriguingly, out of these various DE genes, only 20.3% were found to be upregulated by vAG83 and 52.2% by nvAG83. Conversely, 79.6% of the DE genes were downregulated by vAG83, and 47.7% by nvAG83 (Figure 2A). Moreover, when we compared the overlap in these DE genes (Venn diagram, Figure 3), we found that although 245 genes were modulated by both the parasites, 211 and 228 genes were uniquely modulated by vAG83 and nvAG83, respectively. Further, of the 245 commonly modulated genes, it was found that ~66.0% were downregulated, and 34.0% were upregulated. But of the unique 211 genes modulated by the vAG83, ~95.0% were found to be downregulated and only 5.0% were upregulated. In contrast, of the unique 228 genes modulated by the nvAG83, only ~30.0% were observed to be downregulated and ~70.0% were upregulated. This clearly shows that, in addition to the common genes, vAG83 downregulated many unique genes compared to nvAG83. On the contrary, nvAG83, in addition to the common genes, upregulated many unique genes compared to vAG83. Modulation of these common genes could be explained by the fact that the same parasite, when it loses its virulence upon several passages, may still possess some factors that modulate the host cells in a similar fashion. Overall, there was a significant difference in the host cell gene expression induced by the virulent and the non-virulent parasites. vAG83 employs strategic suppression of macrophage genes at a large scale that may allow them to establish themselves in the host cells (Buates and Matlashewski, 2001). In contrast, the upregulation of many DE genes by nvAG83 parasites might be responsible for their clearance within the host cells (Bhattacharya et al., 2015).

### Validation of the DE Genes by Real-Time PCR

**Figure 4:**
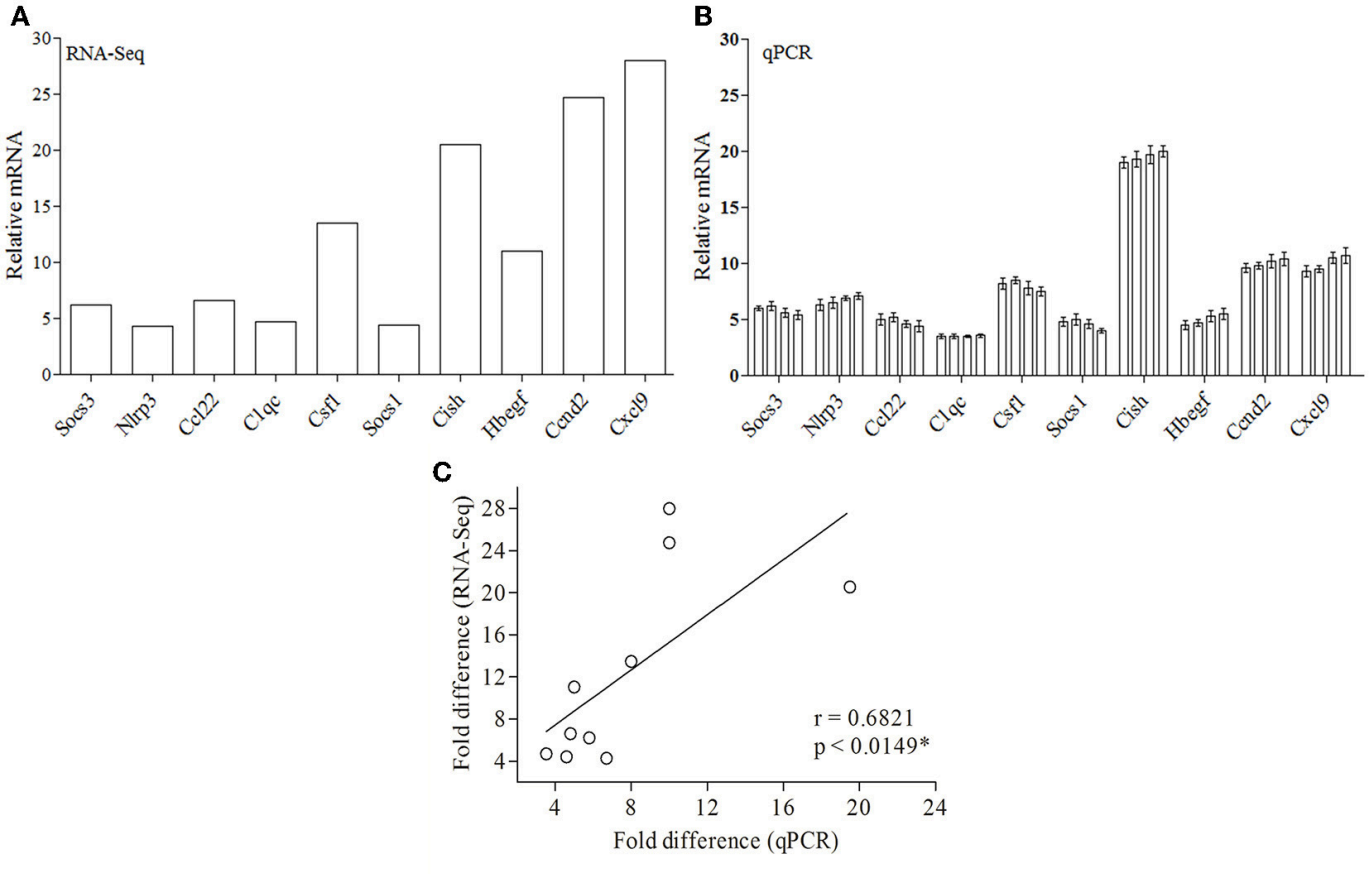
Validation of RNA-Seq-obtained DE genes by qPCR. **(A)** RNA-Seq data for 10 genes are represented in a bar graph. **(B)** The mRNA levels of the same set of genes were measured by qPCR and are represented as the fold change compared to non-infected control cells. **(C)** Fold difference values calculated by RNA-Seq (y axis) for the DE genes in macrophage infected with both parasites correlates to the mRNA expression of these genes using qPCR (x axis).

Since RNA-Seq of a single sample was done for each condition, so as to avoid artifacts and erroneous results, we validated the data obtained from RNA-Seq (Figure 4A) by performing real-time PCR of 10 genes from four biological replicates (Figure 4B). Ten DE genes from macrophages infected with both the parasites, were randomly selected for real-time RT-PCR analysis. The primers of selected genes are listed in Supplementary Table 5. The qRT-PCR results showed a strong correlation with the RNA-Seq-generated data (Pearson correlation coefficients *r* = 0.6973; Figure 4C), thus validating the RNA-Seq results.

### Pathway-Based Enrichment Analyses Depicted a Differential Host Macrophage Response to Infection With vAG83 and nvAG83 Parasites

We next used KEGG pathway enrichment analysis of the DE genes to decipher how the cellular responses evoked against infection by vAG83 and nvAG83 may differ. First, we compared the total number of KEGG pathways, upregulated by both the parasites. We found that vAG83 upregulated only three KEGG pathways, mainly related to immune response and signal transduction, including cytokine-cytokine receptor interaction (Table 3). In contrast, nvAG83 upregulated 34 pathways, of which most are related to immune response and signaling such as cytokine signaling in the immune system, signaling by GPCR, Toll-like receptor signaling and NF-kappa B signaling (Table 4). Similarly, a comparison of the total number of KEGG pathways, downregulated by both the parasites, showed that vAG83 downregulated 13 KEGG pathways, including immune system, signal transduction, gene expression, endocytosis, and phagosome (Table 3). Conversely, nvAG83 downregulated only eight KEGG pathways, including the immune system, signal transduction and transmembrane transport of small molecules (Table 4). This shows that there is a vast difference in the number of KEGG pathways regulated in the host by the two parasites, indicating a huge disparity in the host responses generated against infection by vAG83 and nvAG83. Moreover, this suggests that the macrophages infected with the two parasites may respond to their immediate environment differently. For instance, the nvAG83-infected macrophages (where many pathways of macrophage activation including inflammation are upregulated), seem to become more sensitive to inflammatory signals. Previous reports showed that several inflammatory signaling pathways, including the IL-2 signaling pathway, TNF signaling pathway, Jak-STAT signaling pathway, NF-kappa B signaling pathway, and Toll-like receptor signaling pathway, trigger potent anti-leishmanial immune responses in the host macrophages (Shadab and Ali, 2011). Thus, owing to the immunosuppressive nature of the disease caused by the virulent strain of *L. donovani*, the induction of these pathways by the non-virulent ones highlights the importance of the immune responses against the parasites. Hence, the pathways which were totally shut down by vAG83 seem to have been upregulated in case of nvAG83 infection, which could possibly activate the macrophages to kill the nvAG83 parasites.

**Table 3:** KEGG pathways enriched in DE genes in murine macrophages infected with vAG83.

**Direction of regulation and KEGG pathway**	**Number of DE genes**	**Pathway size**
**KEGG PATHWAY, UPREGULATED**		
Cytokine-cytokine receptor interaction	7	256
Immune system	6	877
Signal transduction	8	1,855
**KEGG PATHWAY, DOWNREGULATED**		
Adaptive Immune System	7	446
Disease	6	605
Endocytosis	6	216
Gene expression	7	703
Hemostasis	7	411
Immune system	14	877
Innate immune system	7	475
mRNA processing	6	451
Pathways in cancer	6	323
Phagosome	7	171
PI3K-Akt signaling pathway	6	348
Signal Transduction	8	1,855
XPodNet—protein-protein interactions in the podocyte expanded by STRING	13	827

*KEGG pathway analysis was carried out to identify pathways related to immune response, signaling and metabolism etc., overrepresented in genes that constitute the mammalian response to vAG83 infection (P-value < 0.01) relative to uninfected controls. Genes that were differentially expressed (DE) by more than 2-fold were used as input with up- and down-regulated genes considered separately. Moreover, pathways filtered based count of DE genes expressed was considered for more than 5 genes. For each enriched KEGG pathway, the number of DE genes assigned to that pathway and the total number of genes in the pathway, are reported*.

**Table 4:** KEGG pathways enriched in DE genes in murine macrophages infected with nvAG83.

**Direction of regulation and KEGG pathway**	**Number of DE genes**	**Pathway size**
**KEGG PATHWAY, UPREGULATED**		
Adaptive immune system	13	446
Adipogenesis	7	133
B Cell receptor signaling pathway	8	156
Chagas disease (American trypanosomiasis)	6	103
Chemokine receptors bind chemokines	6	50
Chemokine signaling pathway	8	183
Class A/1 (Rhodopsin-like receptors)	9	282
Cytokine-cytokine receptor interaction	19	256
Cytokine signaling in immune system	7	192
Disease	7	605
Downstream signaling events of B cell receptor (BCR)	6	107
Extracellular matrix organization	6	226
Fc epsilon receptor (FCERI) signaling	6	154
GPCR ligand binding	10	391
Hematopoietic cell lineage	6	85
Hemostasis	10	411
HTLV-I infection	10	272
IL-2 signaling pathway	7	76
Immune system	20	877
Innate immune system	9	475
Jak-STAT signaling pathway	8	154
Malaria	6	46
Metabolism	7	1,368
NF-kappa B signaling pathway	8	97
Peptide ligand-binding receptors	8	180
PI3K-Akt signaling pathway	9	348
Rheumatoid arthritis	8	82
Signaling by GPCR	11	1,149
Signaling by SCF-KIT	7	124
Signaling by the B Cell Receptor (BCR)	8	133
Signal Transduction	23	1,855
TNF signaling pathway	6	109
Toll-like receptor signaling pathway	8	96
XPodNet—protein-protein interactions in the podocyte expanded by STRING	13	827
**KEGG pathway, downregulated**		
Focal adhesion	6	182
Hemostasis	8	411
Immune system	9	877
Metabolism	9	1,368
PI3K-Akt signaling pathway	8	348
Signal transduction	7	1,855
Transmembrane transport of small molecules	6	496
XPodNet—protein-protein interactions in the podocyte expanded by STRING	12	827

*KEGG pathway analysis was carried out to identify pathways related to immune response, signaling and metabolism, etc., overrepresented in genes that constitute the mammalian response to nvAG83 infection (P-value < 0.01) relative to uninfected controls. Genes that were differentially expressed (DE) by more than 2-fold were used as input with up- and down-regulated genes considered separately. Moreover, pathways filtered based count of DE genes expressed was considered for more than 5 genes. For each enriched KEGG pathway, the number of DE genes assigned to that pathway and the total number of genes in the pathway are reported*.

It was noted that some of the pathways were both upregulated and downregulated by each of the parasites. For example, pathways like the immune system and signal transduction were both up- and down-regulated by vAG83. Similarly, pathways like the immune system, homeostasis, metabolism, signal transduction and the PI3K-Akt signaling pathway, were both up- and down-regulated by nvAG83. This can be explained by the fact that the genes enriched in the up- and the down-regulated pathways are different that may ensue different infection outcomes, based on their functions including positive or negative regulators of infection. For example, in contrast to the genes including Nr4a1, Il1b, Cma1, Socs3, and Dtx1 etc. that were enriched in the upregulated pathways (immune system and signal transduction), the genes of the same pathways when downregulated include Ifi204, Ptprc, Hsp90b1, Actr2, Itga4, Tlr7, Cybb, Trip12, Tax1bp1, Prkcb, Cltc, Hsp90aa1. Apart from these, we also found that some of the pathways downregulated by vAG83 were upregulated by nvAG83—for example, innate immune system, adaptive immune system and disease. Moreover, there were some pathways specifically downregulated by vAG83. These include the adaptive immune system, innate immune system, endocytosis, phagosome, mRNA processing, gene expression and pathways in cancer. The genes enriched in these pathways include Ptprc, Itga4, Trip12, Cybb, Cltc, Prkcb Cav2, Cav1, Met, Csde1, Sf3b1, Eif2ak2, Ddx3x, Pum2, Cpeb4, etc. Previous studies have shown that virulent *Leishmania* parasites hijack the host machinery by employing multiple strategies, including dampening of host immune responses (Gupta et al., 2013), manipulation of endocytosis processes (Verma et al., 2017), inhibition of phagosome biogenesis (Desjardins and Descoteaux, 1997), etc. Altogether, suppression of many key pathways in the host is directly associated with the survival of the virulent parasites. Conversely, their activation is associated with the clearance of the non-virulent parasites.

### Heat Maps Revealed Differential Expression Pattern in the KEGG Pathway-Associated DE Genes Modulated by Virulent and Non-virulent Parasites

**Figure 5:**
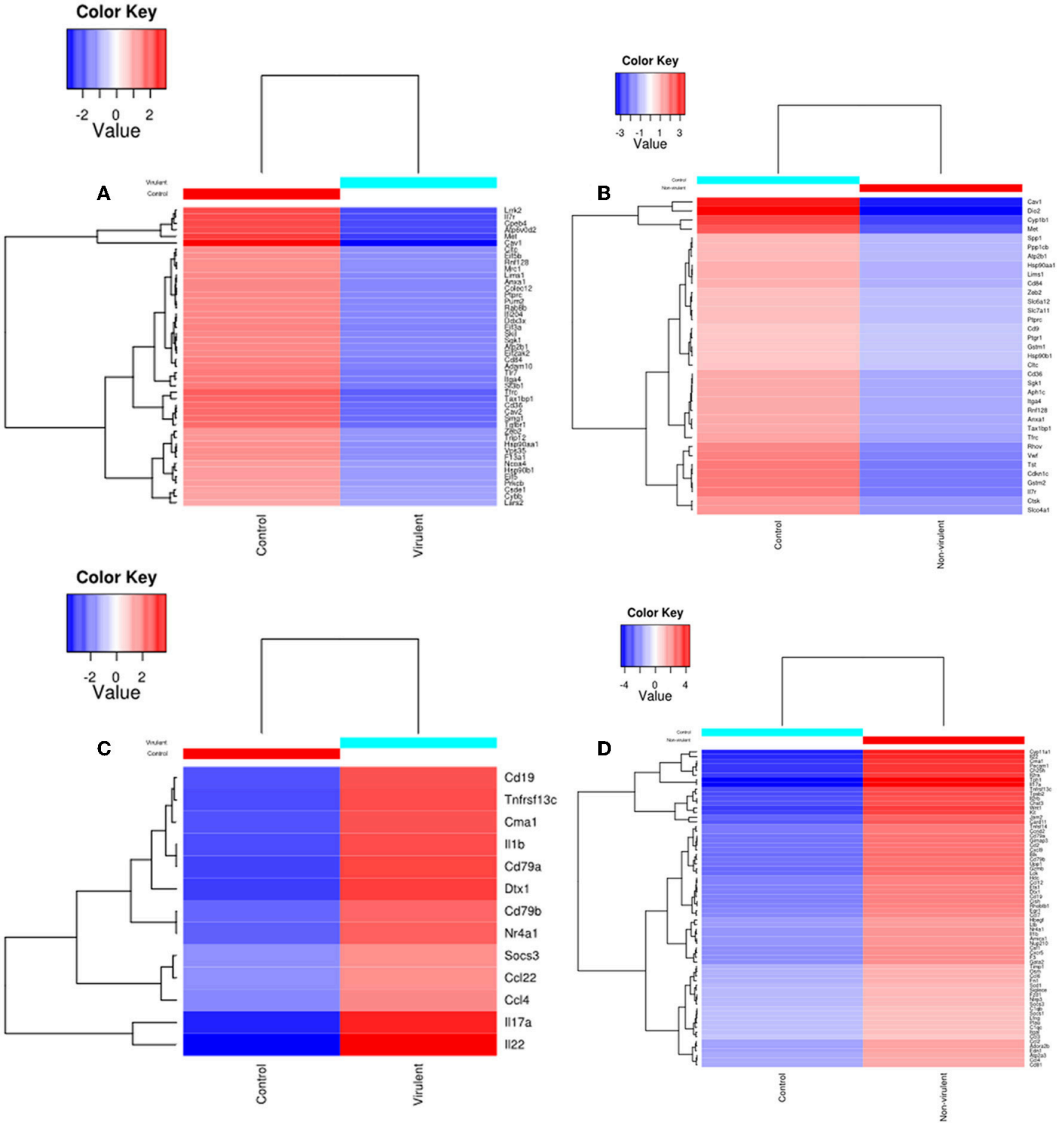
Heat Map of DE genes in infected macrophage compared to control macrophage. The DE genes involved in KEGG pathways downregulated by **(A)** virulent and **(B)** non-virulent parasites are represented as heat maps. Similarly, the DE genes involved in KEGG pathways upregulated by **(C)** virulent and **(D)** Non-virulent parasites are represented.

Heat maps were generated to visualize the level of gene expression induced in the host macrophages by vAG83 and nvAG83. A color code was used to represent high and low levels of expression of the individual genes. These maps were generated from only those genes which constituted the KEGG pathways. This allowed us to achieve a clear picture of how these parasites could produce a different host response, by inducing different levels of gene expression. It was found that vAG83 (Figure 5A) downregulated many genes with significant suppression in their level of expression. In contrast, genes downregulated by nvAG83 (Figure 5B) were smaller in number, with lower suppression compared to that shown by vAG83. Interestingly, a reversed phenomenon was observed for the upregulated genes induced by both the parasites. It was found that vAG83 (Figure 5C) upregulated a smaller number of genes and with a low level of expression. In contrast, nvAG83 (Figure 5D) upregulated many genes with a higher level of expression. Altogether, the heat map reveals that vAG83 and nvAG83 exhibit a differential impact on the number, as well as on the level, of host cell gene expression, which may underlie the differential outcome of the infection.

### Differential Host Macrophage Response to Infection With vAG83 and nvAG83 Involved Biased Inflammatory and Anti-inflammatory Immune Response

Next, the biological functions of the KEGG pathway genes were analyzed, to understand how they could have modulated host cell responses that lead to parasite persistence or clearance (Supplementary Data Sheet 2). We first analyzed the functions of the upregulated genes and found that the host responses evoked by vAG83 and nvAG83 were paradoxical, with clearly biased anti-inflammatory and inflammatory immune responses, respectively. This is because the products of the encoded genes are those which are involved in either enhancing or suppressing the antimicrobial immune responses. For instance, vAG83, which upregulated few inflammatory genes including Il1b, Il17a, and Il22 (Faleiro et al., 2014), also upregulated genes with anti-inflammatory characters, including Ccl22, ccl12, Socs3, and dtx1 (Teixeira et al., 2006). Similarly, nvAG83, in addition to upregulating many inflammatory genes, including Il1b, Il17a, Il22, Ccl2, Ccl3, Ccl4, Ccl12, Cxcl9, Il2ra, Il2rb, and Nlrp3 (Murray et al., 1993; Faleiro et al., 2014), also upregulated genes with anti-inflammatory properties, including Socs1, Socs3, Osm, and Hbegf (Bertholet et al., 2003). Thus, when we analyzed the functions of the downregulated genes, we found that vAG83 suppressed many genes, including Ifi204, Tlr7, Il7r, Nox-2, Eif2ak2, and Prkcb, which are known to be involved in inflammation, macrophage activation and respiratory burst (Nilsson et al., 2006; Shadab and Ali, 2011). However, nvAG83 did not lead to downregulation of too many genes as was observed with vAG83. All these findings are consistent to previous reports, which showed that *Leishmania* parasites could exhibit a differential regulation of macrophage gene expression with mixed immune responses (Gregory et al., 2008). Moreover, they impart a potent and general suppression of macrophage gene expression to establish themselves in the host cells. However, in addition to these, we made an interesting observation that vAG83 downregulated some genes known to be negative regulators of anti-inflammation, like Adam10 (van der Vorst et al., 2015) and Skil (Massagué, 2012). Altogether, these findings indicated that vAG83 tries to maintain an anti-inflammatory environment in the host cells. However, nvAG83 induces an inflammatory environment. This may be the reason that vAG83 persists and nvAG83 gets eliminated in the host cells.

### Modulations in Network Nodes Highlight Their Importance in Parasite Persistence and Clearance in the Host Macrophages

**Figure 6:**
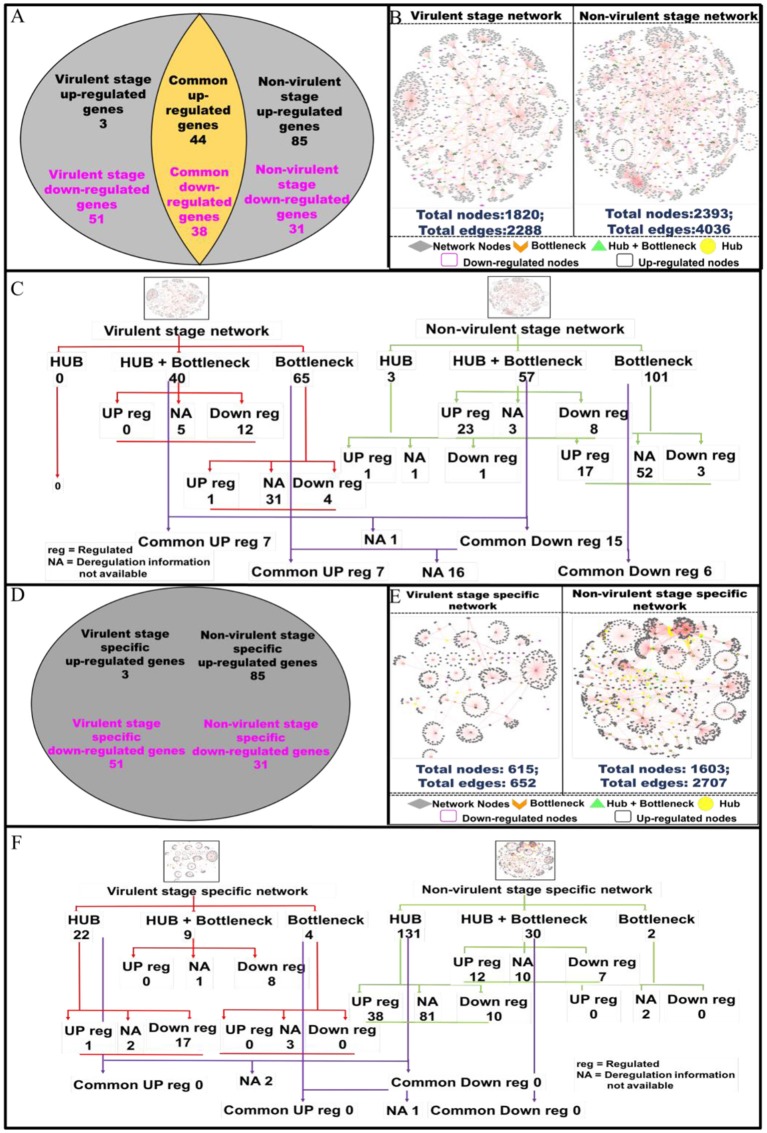
Pictorial representation of the constructed protein-protein interaction network of the deregulated macrophage genes in virulent and non-virulent stages of *L. donovani* infection and analyzing the important nodes of the network. **(A)** Shows the expression status of macrophage genes during virulent and non-virulent stages of *L. donovani* infection, where 54 (3 up-regulated and 51 down-regulated) and 116 (85 up-regulated and 31 down-regulated) genes were found to be expressed during the virulent and non-virulent stages of infection and 82 genes were expressed commonly in both stages. **(B)** Depicts the representation of the constructed virulent and non-virulent protein-protein interaction network. **(C)** Shows the schematic representation of the important nodes of the network. **(D)** Provides the number stage specific deregulated murine macrophage genes at virulent and non-virulent *L. donovani* infection stages. **(E)** Represents the constructed protein-protein interaction network of stage-specific (virulent and non-virulent) deregulated genes. **(F)** Shows the schematic representation of the important network nodes from the stage-specific networks.

Protein-protein interactions (PPI) are essential to almost every process in a cell to maintain its homeostasis (Kuzmanov and Emili, 2013). The proteins involved in such interactions could be of diverse nature, based on their functions viz. enzymes, transcriptional regulators, etc. The complete map of protein interactions occurring in a living organism is called an interactome (De Las Rivas and Prieto, 2012). If the interactome is in any way perturbed, it could lead to alterations in the normal functioning of the cell. Thus, we evaluated how infection with vAG83 and nvAG83 affects host cell interactome by analyzing the macrophage genes that were expressed at *p* < 0.01 (Supplementary Data Sheet 3). These genes were modulated differently by the two parasites. It was found that 3 and 51 macrophage genes (*p* < 0.01) were up- and down-regulated by vAG83, respectively. However, 85 and 31 genes were up- and down-regulated by nvAG83 infection (Figure 6A), respectively. In addition, 44 and 38 common genes were up- and down-regulated respectively, by both parasites. To begin our analysis, we first constructed PPI networks from the total genes, modulated by each of the parasites. We found that the virulent-stage PPI network (VS_PPIN), derived from STRING database, consisted of 2288 interactions with 1820 proteins nodes, whereas the non-virulent-stage PPI network (NVS_PPIN) consisted of 4036 interactions with 2,393 protein nodes (Figure 6B). Based on the graph theory approach, using a topology-based scoring method, important network nodes like hubs and bottlenecks were identified (Figure 6C). “Hubs” are sets of interactive nodes of the network that have a significantly higher amount of connectivity within the network compared to other network nodes (Pang et al., 2016). “Bottlenecks” are network nodes with high betweenness centrality and are key connectors of the sub-networks, thus maintaining the network architecture (McDermott et al., 2009). “Hub-bottlenecks” are the bottlenecks that tend to have high connectivity (Yu et al., 2007). Hub analysis showed that while no hub was found in the virulent-stage network, 3 were found in the non-virulent-stage network. Out of the 3 non-virulent-stage hubs, 1 was downregulated and 1 was upregulated. Interestingly, 40 hub-bottlenecks for the virulent and 57 for the non-virulent stages were found. Overlap of the hub, hub-bottlenecks and bottleneck proteins, identified for virulent and non-virulent stages, are shown in Figure 6C. Overall, the downregulation of many hub-bottlenecks and bottlenecks in macrophages infected with vAG83, indicates a huge collapse in the protein interaction network, restoration of which could otherwise have activated these cells to fight against infection. Meanwhile, in nvAG83-infected macrophages, the upregulation of many hub-bottlenecks and bottlenecks indicates that the overall interactome gets facilitated significantly. This may have activated the host cells to clear off the parasites. Previous study on the responses of macrophages to virulent and attenuated *Mycobacterium bovis* also showed that these two parasites differentially modulate hub and bottleneck genes in the host cells (Killick et al., 2014). We therefore constructed networks based on unique genes, modulated by each of the parasites (Figure 6D). The virulent-stage-specific PPIN (VSP_PPIN) was found to contain 652 interactions comprised of 615 nodes, whereas the non-virulent-stage-specific PPIN (NVSP_PPIN) contained 2,707 interactions, formed by 1,603 nodes (Figure 6E). Figure 6F shows the overlap of the hub, hub-bottlenecks, and bottleneck proteins identified for virulent- and non-virulent-stage-specific networks. Here also, we observed a relatively higher number of downregulated hubs and hub-bottlenecks in the virulent stage network, with upregulation of many hubs and hub-bottlenecks in the non-virulent stage specific network.

Functional and pathway information regarding the deregulated important network proteins are also provided in (Supplementary Data Sheet 4). Molecular function annotation of the virulent-stage-specific important proteins indicates their probable involvement in cytoskeleton structural activity, transcription factor binding, regulation of the RIG-I signaling pathway, membrane trafficking, etc. Further, non-virulent-stage-specific proteins are known to be involved in Wnt-protein binding activity, response to interferon-gamma, inflammatory response, cytokine activity, CXCR3 chemokine receptor binding, CD4 and CD8 receptor binding and CARD domain binding activities. (Supplementary Data Sheet 5). Previous studies have shown that *Leishmania* parasites manipulate actin cytoskeleton (Roy et al., 2014), membrane trafficking (Matte and Descoteaux, 2016), and pathogen-associated molecular pattern signaling, including RIG-I-like receptor signaling (Fernandes et al., 2016), to establish infection in the host cells. However, activation of the inflammatory signals like interferon-gamma (Kima and Soong, 2013), Wnt5a (Chakraborty et al., 2017), and CXCR3 chemokine receptor signaling (Murray et al., 2017), were shown to resist *Leishmania* infection. Altogether, vAG83 significantly downregulated the network nodes, which may help the parasites to evade the host cell's anti-leishmanial immune responses. On the contrary, nvAG83 significantly upregulated the network nodes, which may have activated the host macrophages to kill the parasites.

We next evaluated the modulation in the protein network nodes, which have the highest degree of connectivity (DOC). For this, we analyzed the expression of the top 30 network nodes regulated by vAG83 and nvAG83 (Supplementary Figure 2). Interestingly, vAG83 downregulated many such nodes (25 of 30) compared to nvAG83 (16 of 30). In contrast, nvAG83 upregulated many of the nodes (14 of 30) compared to vAG83 (5 of 30). Together, this suggests that vAG83 not only hampered the physiological expression, but also decreased the number of interactions by downregulating many network proteins, including hub and bottlenecks (Anax1, Hsp90aa1, Psme4, Sf3b1, Smg1, Cav1, Cd36, Hsp90b1, Cybb, Adam10, etc.), leading to the deactivation of the host cells. Conversely, nvAG83 not only upregulated the expression, but also increased the number of interactions by upregulating many network proteins, including hub and bottlenecks (Ccl6, Cxcr5, Edn1, Timp, Kit, etc.), resulting in activation of the host cells.

### vAG83 and nvAG83 Differentially Activated MAPK and PI3K Signaling in the Host Macrophages

**Figure 7:**
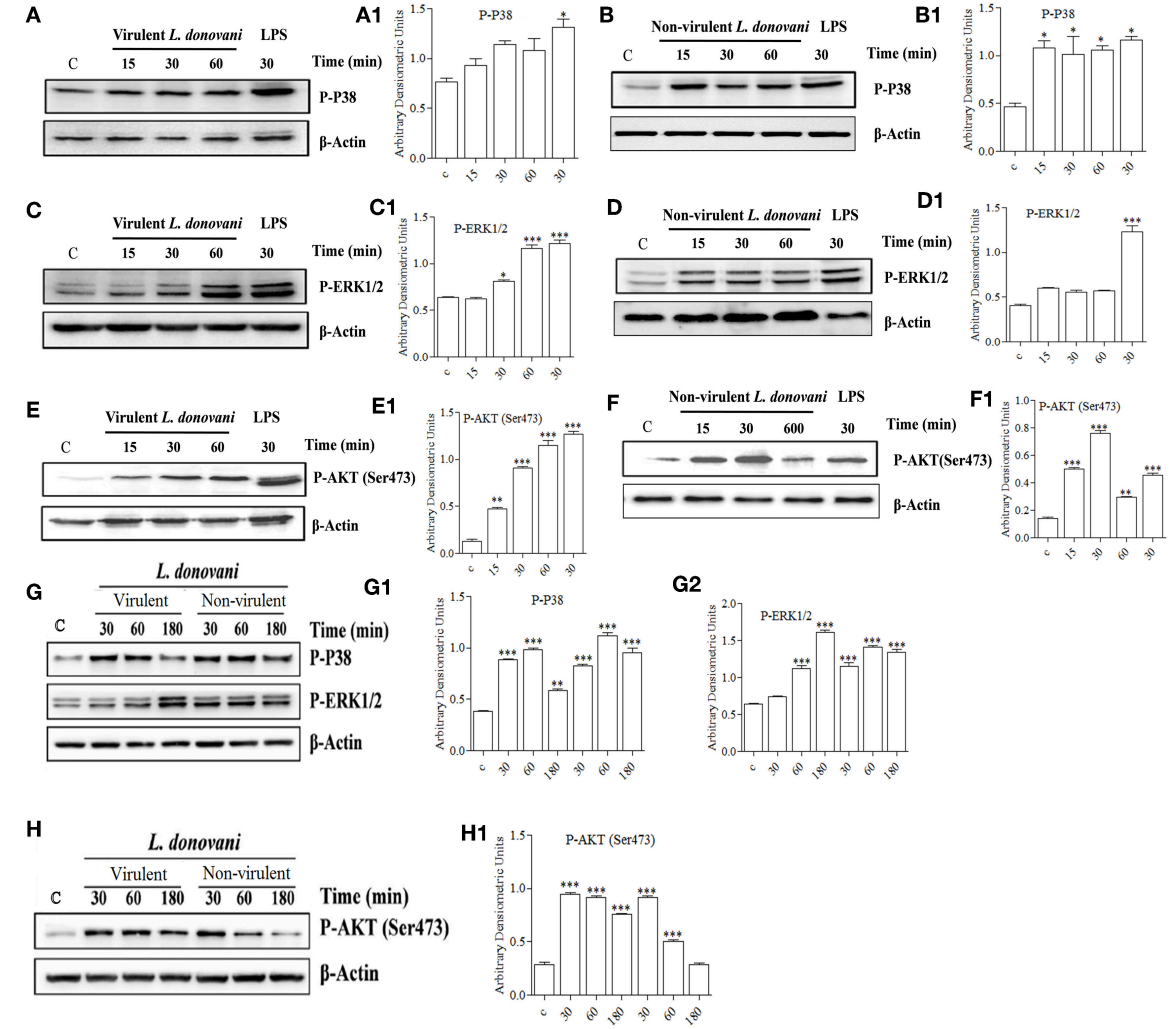
Effect of *L. donovani* infection on MAPK and PI3K activation. Peritoneal macrophages were exposed to virulent and non-virulent *L. donovani* promastigotes for various times as indicated. LPS-treated macrophages were taken as a positive control and non-treated macrophages were taken as a negative control. Whole-cell lysates were prepared and subjected to western blotting using antibodies specific for **(A,A1,B,B1)** phospho-P38 MAPK, **(C,C1,D,D1)** phospho-ERK1/2 MAPK, and **(E,E1,F,F1)** phospho-AKT (ser473). Similarly, cell lysates of BMM exposed to virulent and non-virulent *L. donovani* promastigotes for various times as indicated were probed for **(G,G1,G2)** phospho-P38 MAPK and phospho-ERK1/2 MAPK, **(H)** phospho-AKT (ser473). β-actin was used as loading control for all the blots as described in materials and methods. The figures are representative of 2 independent experiments. Bands were analyzed densitometrically and bar graphs expressing arbitrary units are presented adjacent to corresponding western blots. Error bar represent mean ± S.D., *n* = 2. ^*^P < 0.01, ^**^P < 0.001, ^***^P < 0.0001.

It is well known that the activation of a specific signaling pathway triggers specific sets of gene expression in the cells (Sweeney et al., 2001). In the context of *L. donovani* infection, previous studies have reported that the host cell's signaling is activated in such a way that it allows parasite survival in the host macrophages (Shadab and Ali, 2011). In this study, since we found that vAG83 and nvAG83 induced differential gene expression, we evaluated how these parasites could have manipulated signaling in the host cells. We studied mainly the MAPK and PI3K signaling pathways, whose role in *Leishmania* infection has been reported earlier (Junghae and Raynes, 2002). Through western blot analysis using a specific Ab for phosphorylated form of P38, we showed that vAG83 induced a mild activation of p38 MAPK in the host macrophages as compared to the uninfected control (Figures 7A,A1). In contrast, nvAG83 induced a sustained activation up to 60 min post-infection (p.i.) (Figures 7B,B1). As a positive control, macrophages were stimulated with LPS for 30 min. Studying ERK1/2 MAPK showed that the vAG83 induced ERK1/2 activation in the host macrophages at 30 min p.i., which was enhanced further at 60 min of infection, compared to the uninfected control (Figures 7C,C1). In contrast, nvAG83 induced mild activation of ERK1/2, which remained so until 60 min p.i. (Figures 7D,D1). When we measured the phosphorylation of AKT at Ser473, we found that vAG83 initially induced low activation of AKT (ser 437) as early as 15 min p.i., which increased further at 60 min of infection as compared to the uninfected control (Figures 7E,E1). In contrast, nvAG83 induced AKT (ser 437) activation, which peaked at 30 min p.i. but diminished later on at 60 min of infection (Figures 7F,F1). Similar to murine peritoneal macrophages, BMM were infected with vAG83 and nvAG83 to check if a similar phenomenon was occurring in both the macrophage systems. We found that vAG83 induced P38 activation in the host macrophages until 60 min p.i., which diminished later at 180 min p.i. compared to the uninfected control. However, nvAG83 maintained P38 activation even at 180 min p.i. (Figures 7G,G1). A marked difference in the activation of ERK1/2 MAPK and AKT was observed. vAG83 induced ERK1/2 activation in the host macrophages, which progressively increased, peaking at 180 min p.i. compared to the uninfected cell. In contrast, nvAG83 induced mild activation of ERK1/2 that remained active until 180 min p.i. (Figures 7G,G2). Similarly, vAG83 induced AKT activation as early as 30 min p.i. that remained active until 180 min of infection. In contrast, nvAG83, though inducing AKT activation at 30 min p.i., diminished progressively until 180 min of infection (Figures 7H,H1). Taken together, these results suggest that the two parasites differentially activated MAPK (P38 and ERK1/2) and PI3K signaling both in peritoneal macrophages and BMM, with a similar trend of activation. Interestingly, this differential activation is not linked to an opposite phenomenon of activation and deactivation by the two parasites, but it entails a temporal regulation of the same repertoire of signaling proteins. It has been reported earlier that spatiotemporal activation profiles of the same repertoire of signaling proteins exhibit different gene expression patterns and diverse physiological responses in a cell (Kholodenko, 2006). Hence, the additional time for which a particular kinase remained active before its dephosphorylation seems to produce significant difference in the host cell's gene expression.

The role of ERK1/2 and P38 MAPK in differentially regulating gene expression has been documented previously. It has been shown that ERK1/2 negatively regulates, whereas P38 MAPK positively regulates gene expression (Carter and Hunninghake, 2000). Our finding that vAG83 rather than nvAG83 induced higher activation of ERK1/2 over P38 MAPK indicates a probable strategy employed by the virulent parasite to negatively regulate gene expression and dampen host immune response. Activation of AKT by the virulent *L. donovani* parasite has been shown to inhibit host cell apoptosis (Gupta et al., 2016). Our observation that vAG83 rather than nvAG83 induced higher and sustained activation of AKT in the host cells indicates additional ways of evading host immune responses by the virulent parasites. Why the two parasites impart a differential activation of MAPK and PI3K signaling is not yet clear. There have been reports that the difference in the virulence factors, including cell surface molecules, leads to difference in infectivity of the *Leishmania* parasites (Descoteaux and Turco, 1999). With long-term culture, modifications in such factors, including surface molecules, could happen in nvAG83 (Sinha et al., 2018) which could have triggered different signaling patterns in the host macrophages. Overall, the manipulation of host cell gene expression through specific modulation of MAPK and PI3K signaling by vAG83 and nvAG83 seems to play an important role in the survival or clearance of these parasites in the host cell.

The observation that BMM, as compared to peritoneal macrophages, manifested longer MAPK, and PI3K activation profiles, is not well understood. The peritoneal resident macrophages are un-manipulated cells (Zhang et al., 2008), whereas the bone marrow-derived macrophages are differentiated mature macrophages, which are achieved after differentiating the progenitor cells by adding macrophage colony-stimulating factor (M-CSF) (Austin et al., 1971). Hence, a small disparity in these two macrophage systems can occur which may lead to such difference. Future study can unveil the importance of such difference in *Leishmania* infection, as many sites including bone marrow, liver and spleen, have been shown to be infected with the parasites (Sinha et al., 2015).

### Differentially Expressed *L. donovani* Genes and Gene Ontology-Based Enrichment Analyses for vAG83 and nvAG83 When They Are in the Host Cells

Differential gene expression analyses were carried out for the parasite-specific genes to determine how the expression of the same genes is regulated in the two parasites. It was found that 96 genes were DE between the intracellular amastigotes of vAG83 and nvAG83 at a p value cutoff of < 0.05, which reflected a differential modulation, occurring in these parasites, as they enter the host cells (Supplementary Data Sheet 6). Reasons for not getting a large number of the DE genes could be due to factors including the number of reads, generated for the samples (Mortazavi et al., 2008), the expression level of the genes at this time point of infection, etc. Since these 96 genes were enriched at a lower cutoff value, the functional importance of the genes that showed up beyond this cutoff (data not shown) cannot be denied, and it could modulate parasite responses. Of the DE genes mentioned here, we found ~60% were upregulated and ~40% were downregulated in the amastigotes of vAG83 compared to the nvAG83 (Figure 2B). Moreover, we found that out of all these genes, ~49% (47 of 96) are with uncharacterized function. These hypothetical genes thus constitute a significant portion of the transcriptomic signature of the intracellular amastigotes of *L. donovani* parasites. Future study toward unveiling their function could allow better understanding of their role in the parasites. Moreover, this could boost further the effort in identification of key drug targets, with the possibility that these DE genes might be an integral part of various machineries, operating in the parasites, which are required for their survival.

Next, to evaluate the probable effects of the DE genes on the intracellular amastigotes of the parasites, we performed gene ontology (GO) analysis of these DE genes (Supplementary Data Sheet 7). GO analysis of the up- and down-regulated genes revealed a total of 27 enriched GO categories (Table 5). Of these, 16 GO categories were found to be upregulated in the amastigotes of vAG83 compared to nvAG83. In vAG83, the enriched GO terms (for the upregulated genes) were primarily those that relate to virulence and the survival factors of the parasites. The genes, for example receptor-adenylate cyclases, calpain-like cysteine peptidase, protein kinase, protein kinase-like protein, serine/threonine-protein kinase and mitogen-activated protein kinase, contributed strongly to the GO enrichment results for these parasites (Supplementary Data Sheet 8). Interestingly, these genes were found to be downregulated significantly in nvAG83. The role of receptor-adenylate cyclase in the intracellular amastigotes of *Leishmania* parasites in not known. But in other parasites like *Trypanosoma brucei*, adenylate cyclase is known to inhibit innate immune response of the host (Salmon et al., 2012). Though cysteine peptidase—with higher activity in the intracellular amastigotes compared to the promastigotes of *Leishmania* (Mottram et al., 2004)—is reported, the role of cysteine peptidase with calpain domains is yet to be determined. But in higher eukaryotes, calpains play important roles in calcium-regulated functions such as signal transduction and cell differentiation (Besteiro et al., 2007), indicating that they may have some key roles in the *Leishmania* parasites. Several protein kinases, like CRK3 of the CMGC family and MAPK, have been shown to have essential roles in the proliferation and/or the viability of the *Leishmania* parasites (Naula et al., 2005). Hence, pharmacological inhibition and gene disruption of CRK3 and MAPK, respectively, showed inhibition in the growth and replication of *L. donovani* amastigotes in infected macrophages (Wiese, 1998). Altogether, it indicates that the higher expression of these genes in vAG83 is likely to play a key role in the survival of the parasites in the host cells.

**Table 5:** Gene ontology (GO) categories enriched across virulent to non-virulent *L. donovani* intracellular stages.

**GO ID**	**Go term**	***P-*value**
**UPREGULATED GO TERM IN VIRULENT PARASITE COMPARED TO NON-VIRULENT PARASITE**		
GO:0005524	MF ATP binding	0.003015992
GO:0004514	MF nicotinate-nucleotide diphosphorylase (carboxylating) activity	0.008264463
GO:0004516	MF nicotinate phosphoribosyltransferase activity	0.008264463
GO:0004657	MF proline dehydrogenase activity	0.008264463
GO:0006562	BP proline catabolic process	0.008264463
GO:0009678	MF hydrogen-translocating pyrophosphatase activity	0.008264463
GO:0019357	BP nicotinate nucleotide biosynthetic process	0.008264463
GO:0004198	MF calcium-dependent cysteine-type endopeptidase activity	0.011468442
GO:0005215	MF transporter activity	0.013800439
GO:0015992	BP proton transport	0.016462677
GO:0004427	MF inorganic diphosphatase activity	0.024595158
GO:0009435	BP NAD biosynthetic process	0.024595158
GO:0004672	MF protein kinase activity	0.030803132
GO:0042026	BP protein refolding	0.032662417
GO:0000166	MF nucleotide binding	0.04142298
GO:0004674	MF protein serine/threonine kinase activity	0.048451125
**DOWNREGULATED GO TERM IN VIRULENT PARASITE COMPARED TO NON-VIRULENT PARASITE**		
GO:0051920	MF peroxiredoxin activity	0.000103507
GO:0004601	MF peroxidase activity	0.000342495
GO:0017061	MF S-methyl-5-thioadenosine phosphorylase activity	0.006010518
GO:0004140	MF dephospho-CoA kinase activity	0.011986407
GO:0015937	BP coenzyme A biosynthetic process	0.017927857
GO:0050708	BP regulation of protein secretion	0.017927857
GO:0004402	MF histone acetyltransferase activity	0.023835058
GO:0006414	BP translational elongation	0.023835058
GO:0009116	BP nucleoside metabolic process	0.029708199
GO:0005643	CC nuclear pore	0.035547467
GO:0019843	MF rRNA binding	0.041353051

*GOseq was used to identify enriched GO categories between the virulent and non-virulent intracellular amastigotes at a p-value cutoff of < 0.05. For the difference between both the parasites, up- and down-regulated genes were considered separately. The category for each enriched GO term is indicated (BP, biological process; MF, molecular function; CC, cellular component)*.

Some other genes that were upregulated in the intracellular amastigotes of vAG83 are those that relate to translation machinery, including RNA-binding protein, polyadenylate-binding protein (PABP), ATP-dependent RNA helicase, etc., and protein folding, including Chaperonin HSP60, mitochondrial. This indicates that the protein synthesis in the amastigotes of the virulent parasites is regulated in such a way that may help them to survive and multiply in the host macrophages. Conversely, their downregulation in the non-virulent parasites appears to hamper the production of survival factors that may lead to their clearance.

We found that some of the upregulated GO categories enriched in the intracellular amastigotes of vAG83, as compared to nvAG83, were those that relate to metabolic processes and activities, such as NAD biosynthetic process, nicotinate nucleotide biosynthetic process, nicotinate phosphoribosyltransferase activity, nicotinate-nucleotide diphosphorylase (carboxylating) activity, proline catabolic process and proline dehydrogenase activity. The genes involved in these processes, including nicotinate phosphoribosyltransferase and proline oxidase, were found to be significantly upregulated. Though the role of these genes in *Leishmania* is not yet known, previous studies have highlighted a major role for NAD+ metabolism in the interactions between different pathogens and their host by directly affecting intracellular replication (Kim et al., 2004), virulence expression (Domergue et al., 2005), or pathogen dissemination (Ma et al., 2007). Similarly, proline oxidase, which catalyzes L-proline oxidation to glutamate (Paes et al., 2013) in addition to contributing to the cellular energy supply, plays an important role in the intracellular redox homeostasis and in defense mechanisms against various abiotic and biotic stresses, thus benefiting a broad range of organisms (Ayliffe et al., 2005). Altogether, it thus appears that the host-pathogen interaction, in addition to potentiating metabolic processes in the virulent parasites, also seems to bestow upon them strategies to resist oxidative stress. This, in turn, may allow them to survive and proliferate unrestrictedly in the host cells.

The genes, whose encoded products are known to be involved in transporter activity, proton transport and hydrogen-translocating pyrophosphatase activity, were also upregulated in vAG83. These include ABC1, ABC3, and PPase1. The ABC transporters related to the ABCA, ABCB, ABCC, and ABCG subfamilies have been described in *Leishmania* (Castanys-Muñoz et al., 2008). In fact, ABCC family transporters including MRPA were previously shown to be involved in antimony resistance (Moreira et al., 2013). Moreover, the resistance mechanisms involving increased MRPA expression in the clinical isolates of kala azar were previously implicated (Mukherjee et al., 2007). However, the role of H^+^-PPases in *Leishmania* parasites is not yet known and could be explored in future. Altogether, it appears that the significant upregulation of these genes in the virulent amastigotes is associated with the defense mechanism in the parasites, against the host.

GO terms that were enriched among downregulated genes in virulent amastigotes but upregulated in the non-virulent ones, were mainly related to translation, transcriptional regulation, metabolism and oxidative stress. The product of the DE genes associated with these processes includes 60S acidic ribosomal protein P2, Histone acetyltransferase, Methylthioadenosine phosphorylase, Tryparedoxin peroxidase, etc. Acidic ribosomal protein P2 has been described as a prominent antigen in leishmaniasis. The ribosomal protein P2 from *Leishmania* spp. has in fact been shown to be immunostimulatory (Soto et al., 1995). We also found ribosomal protein S4, a component of the 40S subunit of ribosome, to be significantly upregulated in the amastigotes of nvAG83. Though its role in *Leishmania* parasites is not well understood, yeast Asc1p and mammalian RACK1, which are functionally orthologous core 40S ribosomal proteins, are known to repress gene expression (Gerbasi et al., 2004). A similar function was reported for some other proteins, such as two MYST proteins—Sas2 and Sas3 (Histone Acetyltransferase)—that exhibit transcriptional silencing in *S. cerevisiae* (Sterner and Berger, 2000). Intriguingly, the expression of the putative MOZ/SAS family acetyltransferase was significantly upregulated in the intracellular amastigotes of nvAG83 compared to vAG83. Thus, it reveals that the host-parasite interaction-dependent modulation of the transcriptional regulators in the parasites is vital to the survival of these pathogens in the host cells.

In order to metabolize exogenous and endogenous peroxides, distinct cytosolic and mitochondrial tryparedoxin peroxidases (TXNPx) are present in *Leishmania* as well as *Trypanosomes*. However, some other antioxidant systems, including superoxide dismutases and low-molecular weight thiols, are also present in these organisms (Van Assche et al., 2011). We found that the expression of tryparedoxin peroxidases was upregulated in nvAG83 compared to vAG83. This reflects the fact that despite its higher expression, due to the other factors including loss of virulence, nvAG83 parasites get eliminated by the host cells. Moreover, though earlier studies have shown tryparedoxin peroxidases to overcome oxidative stress when virulent promastigotes enter macrophages (Dillon et al., 2015), the role of other antioxidant systems—including low-molecular weight thiols—should not be denied, which may help virulent parasites to persist within the host cells (Mitra, 2015). Besides tryparedoxin peroxidases, we found that the expression of S-methyl-5′-thioadenosine phosphorylase, MTAP, was also significantly upregulated in the amastigotes of nvAG83 compared to vAG83. MTAP catalyzes the breakdown of S-methyl-5′-thioadenosine (MTA), a major byproduct of polyamine biosynthesis, to adenine and 5-methylthioribose-1-phosphate. Though the role of this gene in *Leishmania* infection is not known, in human cancers including osteosarcoma, malignant melanoma and gastric cancer, MTAP activity acts as a negative regulator of cancer (Miyazaki et al., 2007). Thus, it could be that the upregulated expression of MTA in nvAG83 is a negative feedback mechanism to restrict survival and multiplication of the parasites in the host cells, a supposition that can be explored in future study.

## Conclusion

This study provided a global gene expression pattern of both the host and the parasite, depicting a broad and clear picture of how the changes occurring in these interacting organisms are crucial in determining the final fate of either parasite persistence or clearance. Moreover, this study unraveled many unknown molecules that are associated with these two conditions. This work thus provided valuable information that could serve as a public resource for future efforts toward developing effective anti-leishmanial interventions.

## Ethics Statement

The study was approved by and carried out under the guidelines of the Ethical Committee of the Indian Institute of Chemical Biology, Kolkata. All subjects who participated in this study provided informed consent in writing according to the Indian Institute of Chemical Biology guidelines and approval. The animal experiments were approved by the Animal Ethical Committee (147/1999/CPSCEA) of the institute, according to the National Regulatory Guidelines issued by the Committee for the Purpose of Control and Supervision on Experimental Animals (CPCSEA), under the Division of Animal Welfare, Ministry of Environment and Forest, Government of India.

## Author Contributions

MS, SD, RS, NA, and SC conceived and designed the experiments. MS, RS, SD, MD, and BJ performed the experiments. MS, RS, AB, SC, NA, SD, MA, MM, MK, AT, MKu, and BK analyzed the data. MS, RS, AB, BJ, SK, and NA wrote the paper.

### Conflict of Interest Statement

The authors declare that the research was conducted in the absence of any commercial or financial relationships that could be construed as a potential conflict of interest.

## Supplementary Material

Supplementary Figure 1 Bioinformatics Analysis Workflow. Step-by-step bioinformatics analysis was done for the reads generated for each of the three samples.

Supplementary Figure 2 Top gene nodes that displayed the highest DOC with their expression pattern. **(A)** Top 30 gene nodes modulated by virulent parasite with highest DOC and their level of expression represented as log_2_ fold change. **(B)** Top 30 gene nodes modulated by non-virulent parasite with highest DOC and their level of expression represented as log_2_ fold change.

Supplementary Table 1 Raw data statistics.

Supplementary Table 2 Clean data statistics.

Supplementary Table 3 Alignment Statistics. Read alignment to *Mus musculus* genome.

Supplementary Table 4 Alignment Statistics. Read alignment to *Leishmania donovani* genome.

Supplementary Table 5 Primer sequences used for real-time PCR. Primers for the respective genes were designed using Input3 primer design software and validated using IdT oligoanalyser software and blast software.

Supplementary Data Sheet 1 Gene list for the DE genes in murine macrophage infected with **(A)** virulent and **(B)** non-virulent *L. donovani* parasites. In infected macrophages compared to uninfected control, genes that were differentially expressed (DE) at a *P-*value < 0.05 were used as input with up- and down-regulated genes considered separately.

Supplementary Data Sheet 2 List of DE genes constituting the KEGG pathways enriched in murine macrophages infected with **(A)** virulent and **(B)** non-virulent *L. donovani*. In infected macrophages compared to uninfected control, genes that were differentially expressed (DE) by more than 2-fold at a *P-*value < 0.01 were used as input with up- and down-regulated genes considered separately.

Supplementary Data Sheet 3 List of DE genes in murine macrophage infected with virulent and non-virulent *L. donovani* parasites. In infected macrophage compared to uninfected control, genes that were differentially expressed (DE) at a *P-*value < 0.01, were used as input with up- and down-regulated genes considered separately. The unique genes modulated by **(A)** virulent and **(B)** Non-virulent and **(C)** common downregulated, **(D)** common upregulated genes were listed separately.

Supplementary Data Sheet 4 Pathways, molecular functions and cellular functions of important protein network nodes modulated by each of the parasites. List of Hub and Bottleneck, Bottleneck constructed from Virulent and Non-virulent *L. donovani* infected macrophage genes with their involvement in different pathways, having specific molecular functions and their impact on cellular function including pathogenesis.

Supplementary Data Sheet 5 Pathways, molecular functions and cellular functions of important protein network nodes modulated by each of the parasites. List of Hub-Bottleneck, Hub constructed from Virulent and Non-virulent *L. donovani* infected macrophage genes with their involvement in different pathways, having specific molecular functions and their impact on cellular function including pathogenesis.

Supplementary Data Sheet 6 Gene list for the DE genes in *L. donovani* parasites. In virulent parasite compared to the non-virulent ones, genes that were differentially expressed (DE) at a *P-*value < 0.05, were used as input with up- and down-regulated genes considered separately.

Supplementary Data Sheet 7 Gene ontology for the DE genes in *L. donovani* parasites. In virulent parasite compared to the non-virulent ones, gene ontology analysis of the DE genes at a *P*-value < 0.05, was done to elucidate their probable functions in host macrophage.

Supplementary Data Sheet 8 The DE genes enriched for each GO term in *L. donovani* parasites. In virulent parasites compared to the non-virulent ones, genes that were differentially expressed at a *P-*value < 0.05, were searched for their enrichment in the GO terms to elucidate their probable functions in host macrophage.
