# Supplementary figures and images for: RNA-Seq Revealed Expression of Many Novel Genes Associated With Leishmania donovani Persistence and Clearance in the Host Macrophage
Source: Front Cell Infect Microbiol. 2019 Feb 5;9:17. doi: 10.3389/fcimb.2019.00017 (PMC6370631; doi:10.3389/fcimb.2019.00017)

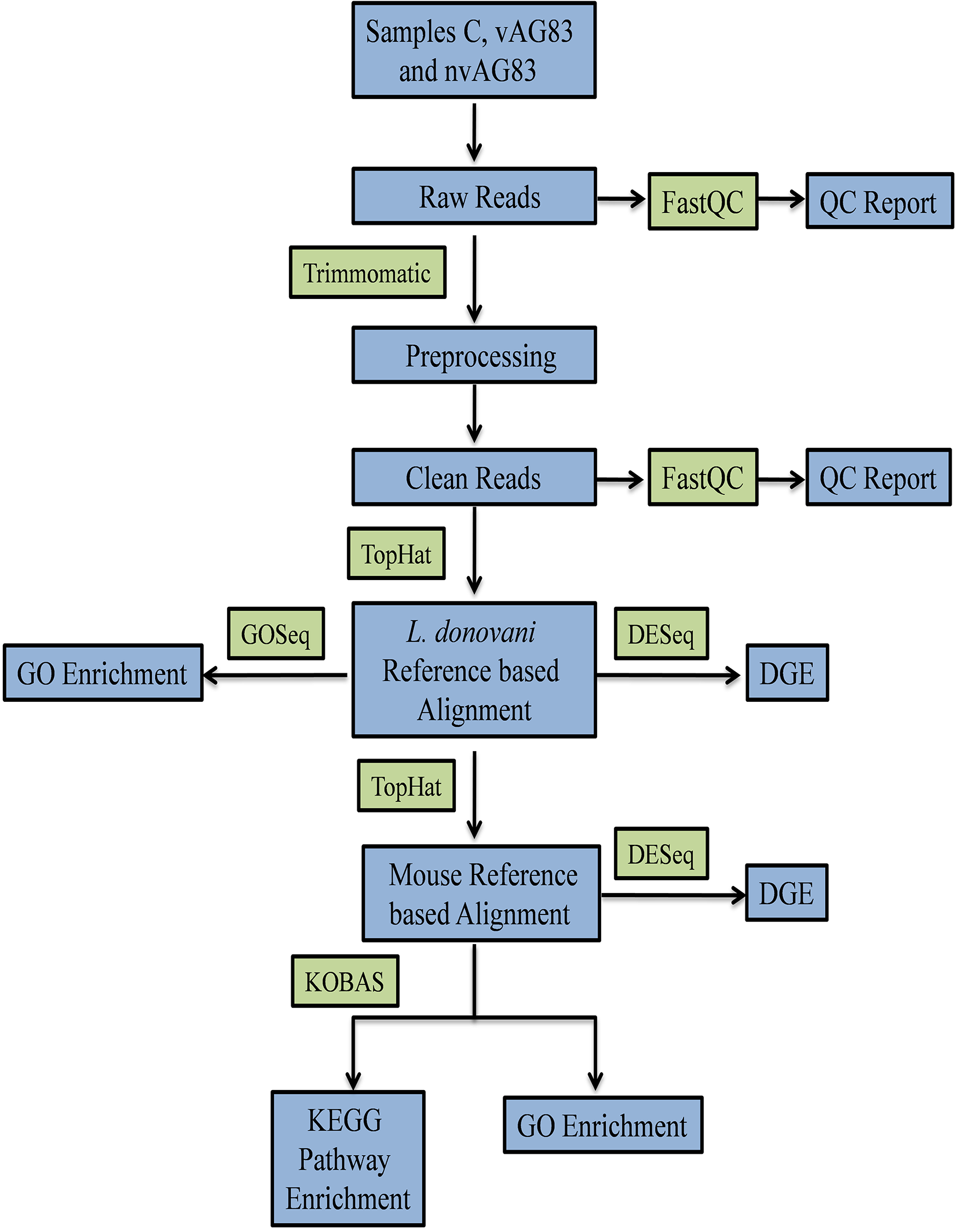

Supplement: Supplementary Figure 1 — Bioinformatics Analysis Workflow. Step-by-step bioinformatics analysis was done for the reads generated for each of the three samples. [file Image_1.TIF]

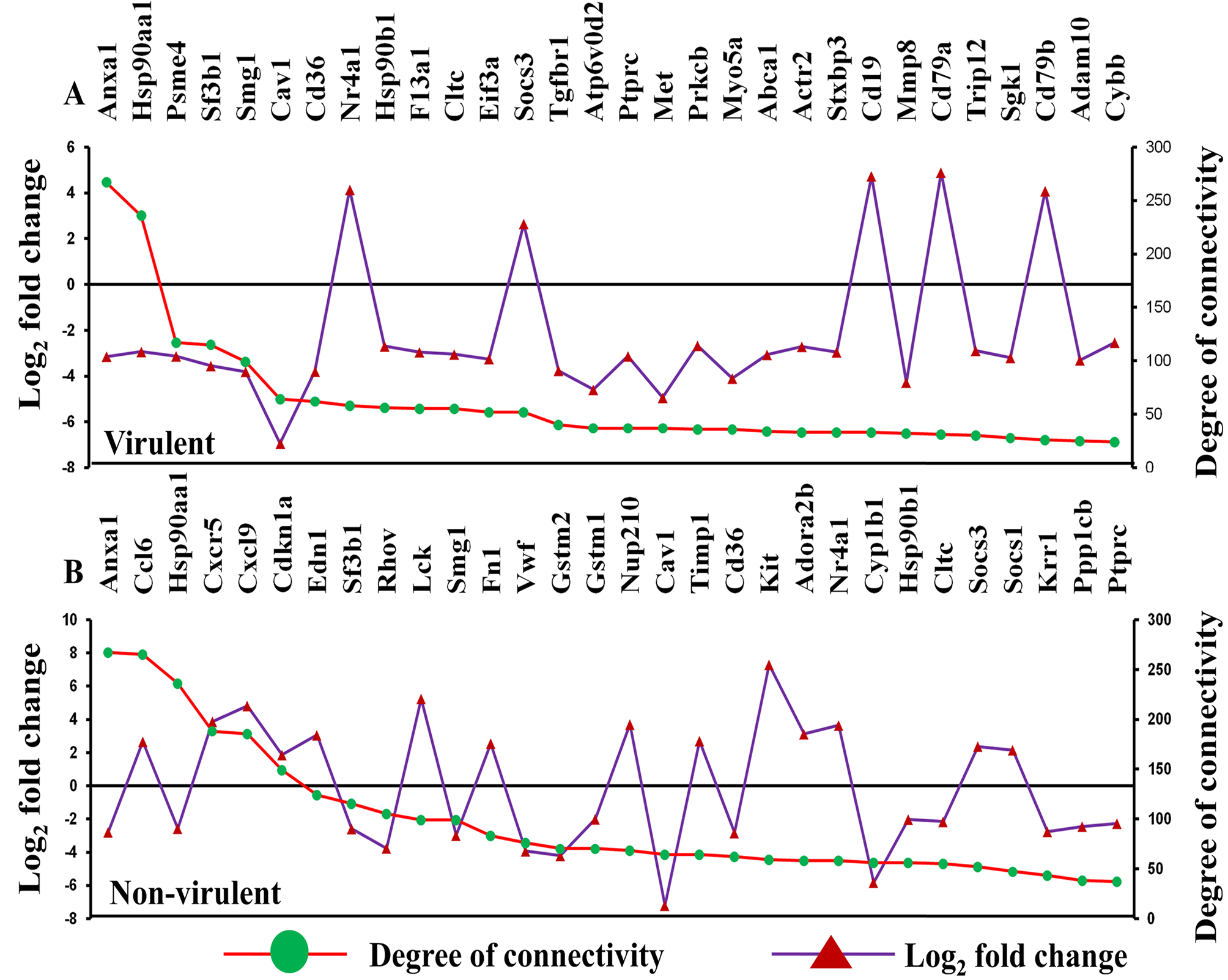

Supplement: Supplementary Figure 2 — Top gene nodes that displayed the highest DOC with their expression pattern. (A) Top 30 gene nodes modulated by virulent parasite with highest DOC and their level of expression represented as log2 fold change. (B) Top 30 gene nodes modulated by non-virulent parasite with highest DOC and their level of expression represented as log2 fold change. [file Image_2.TIF]
