# Supplementary material for: RNA-Seq Revealed Expression of Many Novel Genes Associated With Leishmania donovani Persistence and Clearance in the Host Macrophage
Source: Front Cell Infect Microbiol. 2019 Feb 5;9:17. doi: 10.3389/fcimb.2019.00017 (PMC6370631; doi:10.3389/fcimb.2019.00017)
Supplement: Supplementary Table 1 — Raw data statistics. [file Table_1.docx]

**Supplementary table S1: Raw Data Statistics**

| **Sample Name** | **Number of PE Reads** | **Total Reads (PE x 2)** |
| --- | --- | --- |
| C | 17,270,877 | 34,541,754 |
| A25 | 14,540,412 | 29,080,824 |
| A2nd | 14,552,382 | 29,104,764 |

**Supplementary table S2: Clean Data Statistics**

| **Sample Name** | **Total PE Reads** | **Total SE Reads** | **Total Reads (PE x 2 + SE)** |
| --- | --- | --- | --- |
| C | 16,107,055 | 1,088,042 | 33,302,152 |
| A25 | 13,318,935 | 1,142,783 | 27,780,653 |
| A2nd | 13,629,235 | 856,858 | 28,115,328 |

**Supplementary Table S3: Alignment Statistics**

**Supplementary Table S4: Alignment Statistics**

**Supplementary Table S5:**

**Primer sequences used for Real-time PCR**

| **Target** | **Forward primer (5’-3’)** | **Reverse primer (5’-3’)** |
| --- | --- | --- |
| GAPDH | CATGGCCTTCCGTGTTCCTA | CCTGCTTCACCACCTTCTTGAT |
| Socs3 | GACAGATGAGGCTGGTGAGC | TGTAGCCACCTGGGTGAATC |
| Nlrp3 | CTGCTATCAAGCCCTCCTTC | AGTCACCAAGAGGGAACACC |
| Ccl22 | GTCCTAGGGAGGAGGACCTG | GGGAGGTAGAGGGACCAGAG |
| C1qc | CAACAGCGTCTTCTCTGGTT | TGATAAATGGCCACAGGAAT |
| Csf1 | TCCATCCCTATGAGTGACCA | CCTCCTGGGAATGTCAAAGT |
| Socs1 | CTTAACCCGGTACTCCGTGA | GAGGTCTCCAGCCAGAAGTG |
| Cish | GCAGTAACTTGCCAAGAGGA | TACCAGACCTGACCCTACCA |
| Hbegf | AAAAGGCTGAGGAAGAGCAG | ATGGGGACTGTGACAGCATA |
| CCnd2 | CTAAGACAGGGTGGCTTTCA | GGAGTCCTTTGGGCTTAGAG |
| Cxcl9 | CAGGGAACCCATTTCTCTCTTC | CAGGCTTTGGCTAGTCGTTAT |
